# Cross-generational mechanisms of maternal gut microbiota in modulating offspring autism spectrum disorder risk: from the gut-brain axis to translational challenges in precision interventions

**DOI:** 10.3389/fnagi.2025.1642240

**Published:** 2025-11-05

**Authors:** Dashuang Zhang, Min Zhou, Yudan Qiu, Hong Xu, Hanmin Liu, Yang Liu, Liang Xie

**Affiliations:** ^1^Department of Pediatric Pulmonology and Immunology, West China Second University Hospital, Sichuan University, Chengdu, China; ^2^Key Laboratory of Birth Defects and Related Diseases of Women and Children (Sichuan University), Ministry of Education, Chengdu, China; ^3^The Joint Laboratory for Lung Development and Related Diseases of West China Second University Hospital of Sichuan University and School of Life Sciences of Fudan University, West China Institute of Women and Children’s Health, West China Second University Hospital, Sichuan University, Chengdu, China; ^4^Department of Gynecology and Obstetrics Nursing, West China Second University Hospital, Sichuan University/West China School of Nursing, Sichuan University, Sichuan, China; ^5^NHC Key Laboratory of Chronobiology (Sichuan University), Chengdu, China; ^6^Department of Pediatric Pulmonology and Immunology, WCSUH-Tianfu·Sichuan Provincial Children’s Hospital, Sichuan University, Meishan, China; ^7^Sichuan Birth Defects Clinical Research Center, West China Second University Hospital, Sichuan University, Chengdu, China

**Keywords:** maternal gut microbiota, autism spectrum disorder, gut-brain axis, microbial metabolites, perinatal dysbiosis, fetal neurodevelopment

## Abstract

Autism Spectrum Disorder (ASD) manifests as a group of neurodevelopmental disorders with high clinical and genetic heterogeneity, characterized by core features including social communication deficits, repetitive behaviors, and restricted interests. Current research primarily focuses on genetic variations, immune dysregulation, synaptic dysfunction, and gene–environment interactions. Nowadays, accumulating evidence indicates that maternal gut microbiota dysbiosis, induced by high-fat diets, antibiotic overuse, and urbanization, significantly correlates with abnormal fetal neurodevelopment and increased ASD risk. This review systematically delineates three transplacental mechanisms whereby maternal dysbiosis regulates fetal neurodevelopment: Metabolite-mediated pathways, Immune pathway activation, and Epigenetic reprogramming. Meanwhile, the key translational challenges are highlighted. At last, metagenomics-metabolomics-fetal neuroimaging, Development of microbiota metabolite-treated brain organoids, and Artificial Intelligence-driven (AI-driven) probiotic screening were proposed as research directions in future.

## 1 Introduction

Autism Spectrum Disorder (ASD) is a highly heterogeneous, complex neurodevelopmental disorder. Its core clinical manifestations consist of persistent deficits in social communication/interaction and restricted, repetitive behavioral patterns with narrow interests (Urbonaite et al., 2022). Notably, the global prevalence of ASD has increased significantly, exceeding 1% in some regions (Elvers et al., 2020). This underscores that etiological research impacts not only individual health but also public health.

For a long time, researches of ASD have focused on interactions between genetic susceptibility and environmental factors. There have been excellent reviews for the roles of genetic factors and the interaction of genetic and environmental factors in the pathogenesis in ASD (Grayson and Guidotti, 2016; Cheroni et al., 2020; Masini et al., 2020; Sandin et al., 2014), which are not the major topics of this review. Recently, the gut microbiota—a dynamic community of bacteria (predominant), archaea, viruses, fungi, and protists inhabiting the gastrointestinal tract—and its collective gene pool (the gut microbiome) have transformed ASD research paradigms. With a gene catalog surpassing the host genome, the microbiome is termed the “second genome” (Zhang et al., 2021). It maintains gastrointestinal homeostasis, regulates immune development/function, and reinforces intestinal barrier integrity (Winglee et al., 2017). Its composition is highly individualized, shaped by genetics, age, diet, medications (especially antibiotics), and environment.

Clinical observations frequently report comorbid gastrointestinal dysfunction, such as chronic constipation, diarrhea, and abdominal pain in ASD children. The severity of such symptoms often correlates with core behavioral deficits (Hassib et al., 2023). This association implicates the gut-brain axis—a bidirectional communication network linking the gut microbiome, intestinal mucosa, and nervous system via neural, endocrine, immune, and metabolic pathways [e.g., neurotransmitters, short-chain fatty acids (SCFAs, a type of fatty acid with less than six carbon atoms including acetic acid (acetate), propionic acid (propionate) and butyric acid (butyrate)) (Mann et al., 2024)], tryptophan metabolites, cytokines (Tian et al., 2023). This axis profoundly influences neurodevelopment and behavior.

Microbial colonization begins during critical windows of early-life through maternal-offspring microbial transmission (Amat et al., 2022). This process spans three phases: (1) *Prenatal*: Emerging evidence—though still debated—challenges the “sterile womb” hypothesis, suggesting potential low-biomass microbial transfer occurring through blood or placenta (Hernández-Martínez et al., 2022). (2) *Intrapartum*: Vaginal delivery exposes neonates to maternal vaginal/perianal microbes, such as *Lactobacillus*, *Prevotella* and *Sneathia*, whereas cesarean section results in colonization by skin and environmental taxa, including *Staphylococcus*, *Streptococcus* and *Corynebacterium* (Wu et al., 2017). (3) *Postnatal*: Breastfeeding [conveying microbes/HMOs (human milk oligosaccharides, consisting of multifunctional, unconjugated, and non-digestible glycans) (Dinleyici et al., 2023)], skin contact, and caregiving behaviors further shape the infant microbiome, influenced by maternal diet/health/antibiotics (Choi et al., 2016). These processes train immune development, metabolic programming, and neurodevelopment. The disruptions of these processes are implicated in ASD pathogenesis (Luoto et al., 2010).

In ASD research, the gut-brain-microbiota axis represents a critical frontier. Compelling evidence reveals widespread gut microbial dysbiosis in ASD, characterized by reduced diversity, decreased beneficial symbionts (the microbial organisms mutualistically interacting with the host and the interactions are beneficial to both the host and the microbial organisms, e.g., *Bifidobacterium*), and increased potential pathobionts (members of the microbiota associated with the development of clinical diseases, e.g., some *Clostridium* spp.) (Zhang et al., 2021). Such dysbiosis may drive ASD pathophysiology through: (i) Disruption of intestinal barrier integrity (intestinal hyperpermeability [“leaky gut”]), permitting systemic influx of pro-inflammatory mediators; (ii) Alterations in microbial metabolites (SCFAs, secondary bile acids, neuroactive compounds); (iii) Induction of local/systemic immune inflammation (e.g., elevated pro-inflammatory cytokines like IL-6); (iv) Epigenetic modulation of host gene expression (Tartaglione et al., 2022).

Although causality remains debated (e.g., dietary habits may influence microbiota), recent animal models and microbiota transplantation studies support pathogenic roles for gut microbes in ASD (Kang et al., 2013). This supports probiotic [live microorganisms conferring health benefits at adequate doses; e.g., *Lactobacillus*, *Bifidobacterium*, *Saccharomyces boulardii* (Sun et al., 2024)] and fecal microbiota transplantation (FMT) as translational interventions.

This review summarizes key advances linking gut microbiota to ASD. The conceptual frameworks (gut microbiome, gut-brain axis, microbial transmission) were outlined, the mechanistic hypotheses (barrier dysfunction, microbial metabolites, neuroimmune signaling, epigenetics) were analyzed, the clinical/experimental evidence was evaluated, and the challenges for microbial interventions were discussed. Genetic/epigenetic factors were discussed only where they interact with microbiota as they are not the major topics in this review. Future breakthroughs require multi-omics integration to elucidate microbiota-mediated neurodevelopmental mechanisms and enable personalized therapies.

## 2 The gut microbiome in autism spectrum disorder: the association hypothesis

### 2.1 Landmark historical events

During the clinical observation phase from the 1960s to the 1990s, healthcare workers noted that children with autism spectrum disorder (ASD) frequently exhibited feeding difficulties, constipation, diarrhea, and other gastrointestinal (GI) symptoms. These observations suggested potential dysfunctions within the digestive system in ASD patients (McElhanon et al., 2014). Although the 1998 study by Wakefield and colleagues—subsequently retracted due to scientific misconduct—erroneously linked the measles, mumps, and rubella (MMR) vaccine and intestinal inflammation to ASD onset, its invalid conclusions inadvertently heightened scientific and public awareness of gut-related factors in ASD (Sikora, 2015). This attention indirectly stimulated subsequent researches (Zheng et al., 2020).

The advent of high-throughput sequencing technologies, including 16S rRNA gene sequencing and metagenomics along with metabolomics in the 21st century provided the core technical foundation for large-scale, detailed characterization of gut microbiota composition and functional activity in ASD individuals (Xu et al., 2022; Chang et al., 2024). This facilitated the transition of this field into a period of rapid advancement (Wang X. Q. et al., 2018).

Driven by these technologies, some population studies revealed potential dysbiosis patterns in the gut microbiota of ASD patients. For instance, work by Finegold et al. in 2002 and 2010 reported early observations of increased *Clostridium* species abundance in fecal samples from ASD children (Lu and Claud, 2019; Finegold et al., 2002; Finegold et al., 2010). Subsequently, studies by Kang and colleagues in 2013 and 2017, utilizing large cohorts of ASD children, systematically identified several core features of the ASD gut microbiome: reduced alpha diversity, decreased relative abundance of *Prevotella*, and significant alterations in the *Bacteroidota*/*Bacillota* ratio (Kang et al., 2013; Grochowska et al., 2018). Associations between the abundance of specific bacterial genera and the severity of core ASD symptoms were also observed, providing early evidence for microbiota-behavior links. Critically, their research using the maternal immune activation (MIA) animal model offered experimental support for the biological mechanism whereby maternal infection or inflammatory states might alter offspring gut microbiota and thereby impact neurodevelopment (Liu et al., 2022).

To investigate the potential causal role of gut microbiota in ASD pathophysiology, functional validation approaches were employed. A landmark work by Kang et al. (2019) in 2019 demonstrated that transplantation of fecal microbiota from ASD donors into germ-free (GF) mice recapitulated certain ASD-like behavioral phenotypes, such as social deficits and increased repetitive behaviors. This was accompanied by altered expression of ASD-associated neurodevelopmental genes. This study provided the first direct evidence in a living organism that the ASD gut microbiota possesses functional activity sufficient to influence host neurobehavior, crucially establishing causal evidence for gut microbial regulation of central nervous system function.

As the microbiota compositional features have been increasingly delineated, the researches focus in this field have progressively shifted from descriptive analyses towards mechanistic dissection. Current investigations are deeply exploring several core subjects: the potential neuromodulatory effects of microbial metabolites (e.g., SCFAs, bacterial lipopolysaccharide [LPS], tryptophan derivatives) (Silva et al., 2020; Marć et al., 2022; Cox and Weiner, 2018); the role of compromised intestinal barrier integrity (often termed the “leaky gut” hypothesis) in facilitating systemic access for microbial products or inflammatory mediators, potentially impacting the central nervous system (Kelly et al., 2015); and the contribution of microbiota-dysbiosis-induced aberrant neuroimmune activation to neurodevelopmental disorders (Wang et al., 2023). Collectively, the results of these researches suggest a potential contributory role of complex microbiota-gut-brain axis (MGBA) mechanisms in ASD pathogenesis (Ho et al., 2020).

### 2.2 Key theories

#### 2.2.1 Leaky gut syndrome

The increased intestinal permeability, often termed “leaky gut syndrome,” refers to a pathological state characterized by impaired integrity of the intestinal epithelial barrier (Fiorentino et al., 2016). This dysfunction permits the abnormal translocation of undigested food macromolecules, endotoxins (e.g., LPS), microbial metabolites, and other luminal contents into the systemic circulation (Ruiz-Rodríguez et al., 2022). Investigations within ASD researches have delineated biological features associated with this condition in subsets of affected children (Liu, 2022). Clinical evidence links autism spectrum disorder to gut microbiota dysbiosis and barrier dysfunction, with interventions targeting this axis showing therapeutic potential (Al-Ayadhi et al., 2021); concurrently, broader clinical and preclinical studies implicate this ‘leaky gut’ phenotype in the pathogenesis of diverse neurological disorders through neuroimmune mechanisms (Parodi and Kerlero de Rosbo, 2021). Multiple clinical studies reported aberrant serological markers in specific ASD subgroups (Al-Ayadhi et al., 2021). These frequently included elevated levels of zonulin, a protein regulating intestinal tight junctions (although contradictory findings exist in a minority of studies), and elevated levels of lipopolysaccharide-binding protein (LBP) or endotoxin antibodies (Nalbant et al., 2022). Urine metabolomic analyses further revealed significant alterations in concentrations of gut microbiota-associated metabolites, such as p-cresol sulfate and 3-(3-hydroxyphenyl)-3-hydroxypropionic acid (HPHPA) (Sanctuary et al., 2018). Collectively, this evidence suggests that the increased intestinal permeability and systemic translocation of gut microbial products occur in a subset of ASD individuals. Crucially, these alterations are not universally present across the ASD population, highlighting significant heterogeneity.

The potential biological mechanisms linking increased intestinal permeability to altered neurodevelopment in ASD patients remain to be incompletely elucidated and are subject to ongoing scientific discourse. The researches, primarily based on animal models, *in vitro* studies, and clinical observations, proposed two interconnected (yet unproven causal) pathways. The first is the immune-inflammation pathway hypothesis: translocation of microbial-associated molecular patterns such as LPS may activate circulating immune cells like monocytes and macrophages, eliciting a state of low-grade systemic inflammation (Tucureanu et al., 2018). Subsequently, pro-inflammatory cytokines such as IL-6, IL-1β and TNF-*α* released in this response could impact the central nervous system (CNS) via mechanisms including transport across the blood–brain barrier (BBB) and vagal nerve signaling (Morris and Maes, 2014). In animal models, such peripheral inflammation and the ensuing neuroinflammation, for example microglial activation, disrupt synaptic pruning, neurotransmitter balance, and neurogenesis. Within the ASD context, this mechanism represents a biologically plausible hypothesis; however, it is essential to emphasize that the precise multifactorial etiology of ASD remains undefined. The second is the neuroactive substance hypothesis: under conditions of intestinal hyperpermeability, gut microbiota-derived metabolites—certain organic acids—that potentially influence GABAergic signaling or tryptophan-serotonin metabolic pathways may gain aberrant access to the systemic circulation (Boccuto et al., 2013). This hypothesis, however, contains several points of contention. For instance, whether microbially produced GABA can efficiently cross the BBB and attain concentrations sufficient to directly modulate CNS function is questionable; indirect effects, such as modulation of host metabolic pathways, might be more relevant. Conversely, dysregulation of tryptophan metabolism is a hot research focus strongly implicated in neuropsychiatric disorders, including ASD.

Key limitations impede a deeper understanding of the role of intestinal hyperpermeability in ASD. The primary challenge is establishing causality: Existing evidence linking this condition to ASD largely derives from cross-sectional studies (Liu, 2022). Thus, it remains difficult to discern whether increased intestinal permeability and associated dysbiosis are primary drivers of ASD pathogenesis, or if they represent secondary consequences of other ASD-related factors such as restricted dietary patterns, inherent genetic variations, or downstream neurobehavioral alterations affecting gut function. A large-scale metagenomic study by Yap et al. (2021) (n = 247) revealed only a very weak direct association between ASD and gut microbial structure, with *Romboutsia timonensis* being the only species showing statistical significance, albeit with a small effect size. The study further indicated that the observed differences in the gut microbiome of children with ASD are more likely a consequence of their behavioral characteristics (such as restricted dietary preferences and low intake diversity) rather than a causal factor in the development of ASD. However, there are still other factors such as the mode of maternal delivery, breastfeeding, genetics, and drugs attributed to the changes of the gut microbiota in children (Takyi et al., 2025). Furthermore, a meta-analysis (Gao et al., 2025) suggested that microbiome-targeted interventions could have a mild positive effect on improving behavioral symptoms in individuals with ASD, but the overall improvement in gastrointestinal symptoms did not reach statistical significance. This conclusion is limited by the heterogeneity and methodological quality of existing studies, underscoring the need for more rigorously designed clinical trials for validation. Overall, alterations in the gut microbiota of children with ASD result from the interplay of multiple factors, including genetics, perinatal influences, dietary patterns, medication use, and immune-metabolic abnormalities. The current debate highlights the complexity of the etiological mechanisms underlying ASD and suggests that future research should place greater emphasis on the role of intrauterine environment and early developmental factors. Well-designed longitudinal studies, particularly cohorts initiated in early development, are critically needed to delineate the temporal sequence and potential causal relationships. Secondly, there is an issue of biomarker specificity: elevated serum levels of zonulin, LBP, and other markers are not unique to ASD (Heidt et al., 2023; Kim et al., 2023; Ohlsson et al., 2017). Similar elevations are observed in other gastrointestinal disorders (e.g., celiac disease, irritable bowel syndrome) and various systemic inflammatory states, limiting their utility as specific diagnostic or subtyping tools for ASD. Lastly, there is limited therapeutic evidence: interventions targeting gut microbiota modulation, such as specific probiotic formulations or FMT, have reported improvements on behavioral scales (e.g., Autism Behavior Checklist, ABC score) in some underpowered, non-blinded, or open-label studies (Prosperi et al., 2022). Nevertheless, these preliminary findings currently lack validation through high-quality, large-scale, randomized, double-blind, placebo-controlled trials (RCTs). The clinical efficacy, identification of responsive subgroups, and long-term safety of such interventions require rigorous evaluation (Zhang et al., 2022).

Given this complex evidentiary landscape, the prevailing scientific consensus posits that, within the current knowledge framework, increased intestinal permeability is not considered a direct etiological factor for ASD. A more precise conceptualization positions it as a potential “environmental trigger/perpetuating factor” or “disease modifier,” acting primarily within specific ASD subgroups harboring particular genetic susceptibilities (Zheng et al., 2020). It may contribute to neurodevelopmental deviation or exacerbate behavioral symptoms through the aforementioned immune-inflammatory and neuroactive substance pathways, acting upon an existing genetic predisposition (Fiorentino et al., 2016; Rylaarsdam and Guemez-Gamboa, 2019; Zhu et al., 2020). To overcome current limitations and precisely evaluate its pathological significance and potential therapeutic relevance, future research could integrate multi-omics approaches along the gut-brain axis with rigorously designed, prospective, large-scale clinical cohort studies (Takyi et al., 2025). This integrated strategy is essential to definitively elucidate the causal mechanisms of increased intestinal permeability within specific ASD endophenotypes and provide an evidence base for personalized interventions.

#### 2.2.2 Metabolite-neuronal pathway validation

The gut microbiota ecosystem produces diverse metabolites through fermentation of substrates like dietary fiber, some of which exhibit confirmed or suspected neuroactive potential (Miri et al., 2023). These metabolites can mediate gut-brain axis communication through multiple routes, such as the circulatory system, direct vagus nerve transmission, or the enteroendocrine cell-vagus nerve pathway, indirectly influencing central nervous system (CNS) function and development (Kasarello et al., 2023). Key microbial metabolic pathways and their potential roles in ASD are now widely investigated.

SCFAs, the core end-products of dietary fiber fermentation, are primarily generated by specific strains within the Bacteroidetes and Firmicutes phyla (e.g., *Faecalibacterium prausnitzii*, *Roseburia* spp. *luojiarufa junshu*—*Luojiaru Bacteria Genus*), predominantly including acetate, propionate, and butyrate (Chang et al., 2019; Al-Qadami et al., 2022). Butyrate plays multiple beneficial roles in gut health: serving as the preferred energy source for colonic epithelial cells, which is crucial for maintaining intestinal barrier integrity (Al-Qadami et al., 2022; Lee et al., 2021). Furthermore, butyrate’s anti-inflammatory properties and inhibition of histone deacetylases (HDACs) suggest potential regulation of neural plasticity via epigenetic mechanisms (McClung and Nestler, 2008; Dash et al., 2009). Notably, several studies reported decreased abundance of butyrate-producing microbiota with key functional roles (e.g., *F. prausnitzii pulasuojun*—*Pulasou Bacteria*) in fecal samples from individuals with ASD, implying impaired butyrate production might contribute to ASD pathogenesis (Retuerto et al., 2024). Conversely, the neurobiological effects of propionic acid (PPA) exhibit significant complexity and dose-dependence (Nankova et al., 2014; Le Poul et al., 2003). While PPA acts as an important energy substrate and signaling molecule at physiological concentrations, neurotoxic evidence from animal models—primarily involving non-physiological high-dose intracerebroventricular or intraperitoneal injections—clearly demonstrates its ability to induce core ASD-like behavioral phenotypes in rodents, including social deficits, increased stereotypy/stereotypic movements, and sensory processing abnormalities (Choi et al., 2018). Potential mechanisms identified in these models involve mitochondrial dysfunction, aberrant neurotransmitter release—dopamine and glutamate—exacerbated oxidative stress, microglial activation, enhanced neuroinflammation, and aberrant epigenetic regulation (Choi et al., 2018; Nankova et al., 2014; Csoka et al., 2024). Critical controversy remains, however, regarding whether endogenous physiological concentrations or the mild PPA elevation observed in some ASD cohorts suffice to reach significant neurotoxic thresholds within the human CNS (Le Poul et al., 2003). Crucially, the highly inconsistent findings on SCFA profiles in stool or plasma from ASD populations, reflected substantial sample heterogeneity and potential influences of sample type and analytical methodologies (Liu et al., 2019). Moreover, certain behavioral effects of injected PPA were not fully replicated in female rodent models, highlighting the need for caution regarding sex differences when extrapolating model data to humans, especially given ASD’s marked male bias (Kamalmaz et al., 2023).

Beyond SCFAs, the tryptophan metabolic pathway constitutes another critical node linking the gut microbiota to host neural function. As an essential amino acid, tryptophan serves as the precursor for the key CNS neurotransmitter serotonin, which regulates mood and cognition, and melatonin, which regulates for sleep–wake cycles. Gut microbiota (e.g., *Clostridium* spp.) profoundly influence tryptophan metabolism: they directly utilize it for bacterial protein synthesis and convert it to indole derivatives (Wang G. et al., 2024). Importantly, a substantial proportion of dietary tryptophan is metabolized via the host kynurenine pathway (KP) (Munn and Mellor, 2016). Under pro-inflammatory conditions, which are triggered by diverse etiologies, indoleamine 2,3-dioxygenase (IDO) activity is significantly upregulated in host cells—immune cells, enterocytes and hepatocytes—and shunts tryptophan towards kynurenine (KYN) synthesis (Munn and Mellor, 2016). This appears congruent with observations of reduced plasma tryptophan and elevated KYN/tryptophan ratios in some ASD cohorts, indicating potentially widespread IDO activation mediated by inflammatory status. CNS exposure to KP metabolites is critical, as distinct pathway metabolites exert mutually antagonistic neural effects: further KYN metabolism generates the excitotoxic quinolinic acid (QUIN), an N-methyl-D-aspartate (NMDA) receptor agonist inducing excitotoxicity and oxidative stress (Guillemin et al., 2007); concurrently, neuroprotective kynurenic acid (KYNA) is produced, acting as an antagonist at NMDA and α7-nicotinic acetylcholine receptors (Guillemin et al., 2007). Some ASD studies suggested the evidence for upregulated IDO pathway activity and metabolic imbalance, which potentially favors neurotoxicity or excitotoxicity, in affected individuals, and these alterations showed some correlation with clinical symptom severity. However, the core causal mechanisms by which tryptophan-KP alterations drive ASD neuropathology and their precise CNS targets require further elucidation (Savino et al., 2020).

Finally, other microbially-derived metabolites with potential neuroactivity are of growing research interest. For instance, p-cresol, produced by certain *Clostridia*, transforms into its sulfate derivative (p-Cresyl sulfate, PCS) upon host sulfation (Harrison et al., 2021); PCS has been reported enriched in urine from some ASD children (Osredkar et al., 2023). Although its toxicity mechanisms remain incompletely defined, PCS is hypothesized to potentially exert neuroactive effects via interference with critical sulfation pathways or mitochondrial impairment (Mueller et al., 2018); however, current evidence favors its influence on neurodevelopment primarily through complex immune modulation, with direct significant neurotoxicity currently lacking definitive support. Furthermore, secondary bile acids (SBA), generated from host primary bile acids by gut bacterial modification, act as the key signaling molecules (Tie et al., 2023). By activating receptors such as the farnesoid X receptor (FXR) and G protein-coupled bile acid receptor 1 (TGR5/GPBAR1), SBAs regulate host metabolism and inflammation (Tie et al., 2023; Wang Y. et al., 2024). Theoretically capable of mediating brain function via vagal afferent signaling or neuro-immune crosstalk, SBAs represent an emerging research area in gut-brain communication (Kim et al., 2016). Preliminary animal behavioral studies and limited human data suggest associations between altered SBA profiles and certain neurobehavioral traits; however, their specific roles and causal involvement in ASD await systematic investigation and robust evidence.

## 3 External factors influencing maternal gut microbiota and offspring neurodevelopment

**Figure 1:**
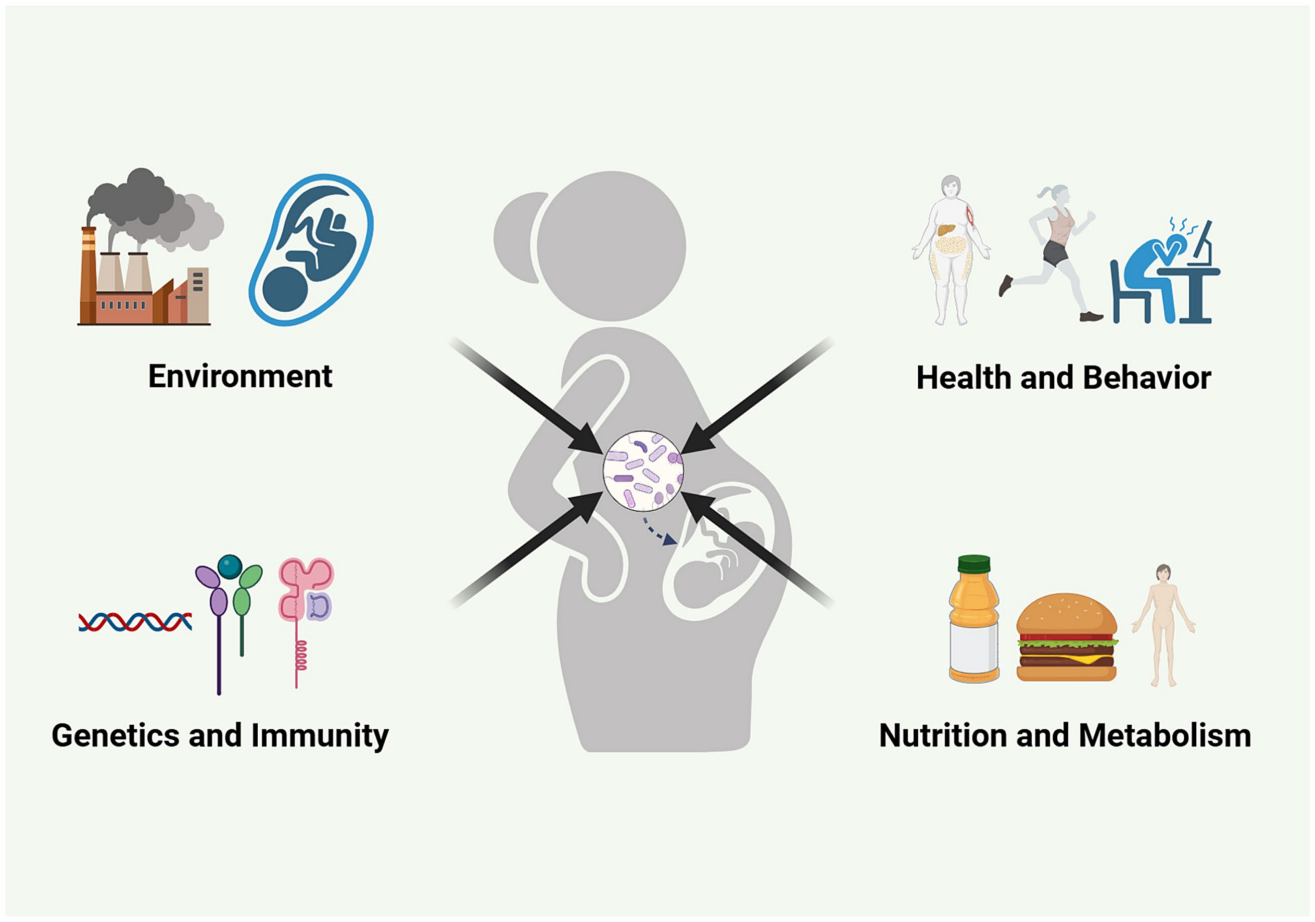
Integrative impact of external factors on maternal gut microbiome and offspring neurodevelopment. Environmental, maternal, genetic, and nutritional factors converge to shape the maternal gut microbiome, which in turn modulates fetal neurodevelopment through metabolic, immune, and epigenetic mechanisms, influencing ASD susceptibility (created in BioRender; Xie, 2025, https://BioRender.com/0xsjbrb).

Previous studies have suggested that several external factors could attend in offspring neurodevelopment by affecting maternal gut microbiota (Figure 1).

### 3.1 Environmental factors

Epidemiological studies confirmed that prenatal exposure to environmental pollutants such as PM_2.5_ and bisphenol A and alterations in delivery modes significantly impacted maternal gut microbiota dynamics (Wang W. et al., 2018). This manifested as increased relative abundance of specific bacterial genera such as *Ruminococcus* and *Staphylococcus* and perturbations in key metabolic pathways such as arachidonic acid metabolism (Filardo et al., 2022). Observational data further revealed statistical associations between such dysbiosis and offspring outcomes including cognitive developmental delay and ASD risk, with pollutant effects exhibiting dose-dependency (Agathokleous et al., 2022). However, existing evidence cannot establish direct causal relationships between individual microbial taxa/metabolic pathways and neurodevelopmental outcomes (Matsuyama et al., 2022).

Biologically, maternal dysbiosis affects fetal neurodevelopment through three principal mechanisms: (i) Microbial metabolites (e.g., SCFAs) regulate brain-derived neurotrophic factor expression via vagus nerve activation or central nervous system GPR41/43 receptors (Kasarello et al., 2023); (ii) Immune-gut-brain axis-mediated Th1/Th2 imbalance induces maternal-fetal interface inflammation, disrupting fetal blood–brain barrier development and triggering neuroinflammatory pathways (Zha et al., 2022; El Ahdab et al., 2021); (iii) Gut microbial biosynthesis of neurotransmitter precursors—5-hydroxytryptamine and *γ*-aminobutyric acid—exerts developmental programming effects (Park and Im, 2022).

Animal models provide critical validation: Probiotic interventions enhanced offspring hippocampal neuroplasticity and improve cognitive performance (Romo-Araiza et al., 2023). Experimental simulations of PM_2.5_ or bisphenol A exposure successfully replicated human phenotypes including reduced maternal microbial diversity and offspring neurodevelopmental abnormalities (Liu, 2018; Senaldi and Smith-Raska, 2020). Current research faces methodological challenges: interindividual heterogeneity in baseline microbiota impedes intervention assessment (Louis et al., 2016); observational studies struggle to exclude confounders, with path analysis indicates that approximately 32% of PM_2.5_ effects may be microbiota-mediated, though weighting depends on model assumptions (Senaldi and Smith-Raska, 2020); additionally, traditional dose–response models inadequately resolve nonlinear exposure patterns, such as short-term pollutant peaks, thereby limiting the generalizability (Hoel and Portier, 1994).

Translational strategies prioritize optimizing environmental exposure thresholds and developing microbiota-targeted interventions. Cross-generational transplantation experiments demonstrated that maternal microbiota modulation partially rescued pollutant-induced teratogenic effects (Hassib et al., 2023). Given substantial interindividual variation in circulating SCFAs during pregnancy (coefficient of variation [CV] = 62%) and host genetic regulation, future researches could integrate exposomics with multi-omics technologies (Hsu et al., 2021). This integration will enable construction of interactive network models spanning environmental exposure dynamics, microbiota succession, and neurodevelopmental trajectories to advance precision prevention (Deek et al., 2024).

### 3.2 Maternal health and behavioral determinants

Epidemiological studies indicated that maternal metabolic status (Krakowiak et al., 2012), nutritional intake patterns, and stress exposure during gestation were associated with offspring risk of ASD. Specifically, maternal metabolic disturbances (e.g., obesity or diabetes) were found to increase ASD risk [reported odds ratios [ORs] = 1.6–2.3 (Krakowiak et al., 2012; Li et al., 2016)], with evidence suggesting a dose-dependent trend. These conditions may impact neurodevelopment through elevated circulating proinflammatory cytokines, such as IL-6 and TNF-*α*, and impaired fetal microglial differentiation (Li et al., 2016).

Animal models demonstrated that high-fat diet (HFD) exposure during pregnancy activated placental inflammatory pathways, leading to aberrant amygdala-prefrontal circuit development (Hill et al., 2015). In specific models, this correlated with an 89% increase in ASD-like behaviors. Maternal deficiencies in key nutrients, such as iron and folate, were linked to compromised hippocampal synaptic plasticity and dopaminergic dysfunction (Bastian et al., 2016). Additionally, prenatal stress-induced glucocorticoids might dysregulate offspring hypothalamic–pituitary–adrenal (HPA) axis reactivity via epigenetic modifications, potentially elevating comorbid anxiety risk in ASD (Hill et al., 2015).

The intricate gut microbiota-host metabolic-immune crosstalk is considered to be the biological basis. Microbiota-derived metabolites such as butyrate and kynurenine may indirectly influence fetal neurodevelopment through histone-modifying enzymes like HDACs by affecting glial differentiation (Yan et al., 2012). Animal evidence supports these mechanisms: antibiotic-induced maternal microbiota depletion caused cerebellar axonal deficits and motor dysfunction in offspring; HFD-exposed dams produced offspring with reduced hippocampal neurogenesis and social deficits; and maternal immune activation via polyinosinic-polycytidylic acid (poly(I: C)) reduced cortical synaptic density, with IL-17A identified as a key mediator.

Notably, targeted interventions (e.g., *Bacteroides fragilis* or *B. thetaiotaomicron* transplantation; multispecies probiotics) partially attenuated these abnormalities in models, supporting microbiota-mediated effects. A phase II trial reported potential language improvement after vaginal microbiota transplantation (VMT) (Wrønding et al., 2023), but long-term neurological safety requires systematic follow-up. Future research must establish ethnically diverse maternal-child cohorts with lifelong tracking to bridge mechanistic insights and clinical prevention.

Current evidence faces significant constraints. Human studies predominantly involve European-ancestry populations (representing approximately 78% of participants in major cohorts) (Pérez-Morales et al., 2024), leading to limitations in generalizability. Furthermore, critical confounders, such as environmental toxin exposure, remain inadequately controlled for in analyses (Keller, 2014; Hernandez et al., 2019). While emerging, robust dose–response data for specific probiotic or prebiotic strains are still scarce (Rees et al., 2002). Translation of research findings into clinical practice requires optimized strategies. Promising avenues include combinatorial approaches; for example, combining high-fiber diets with select prebiotics that modulate *Roseburia* abundance and glutamate transporters might enhance therapeutic efficacy. Additionally, the development and validation of novel biomarkers could significantly improve patient risk stratification, as evidenced by certain composite models achieving promising receiver operating characteristic (ROC)-area under the curve (AUC) values of 0.87 (Li et al., 2019).

### 3.3 Genetic and immunological factors

Epidemiological studies indicated that the maternal genetic background might interact with the gut microbiota, collectively influencing offspring risk for ASD (Rees et al., 2002; Li et al., 2019). Cohort studies revealed that maternal polymorphisms in *IL10* can affect IL-10-mediated immunosuppressive function (Rees et al., 2002). Population-based research further identified that such polymorphisms were accompanied by altered abundance of specific maternal gut microbiota members, such as the family *Alcaligenaceae* and the genus *Acinetobacter* (Li et al., 2019). This gene-microbiota interaction pattern is hypothesized to potentially affect the fetal immune microenvironment via microbial metabolites.

A research on mother-infant microbiota vertical transmission demonstrated that the children later diagnosed with ASD frequently exhibited reduced abundance of gut bacterial genera involved in SCFAs production, such as *Prevotella* (Li et al., 2019). This reduction correlated with dysregulated Th17/Treg immune balance and was associated with neuroinflammation (Li et al., 2019; Blagonravova et al., 2021; Bik et al., 2018).

As to biological mechanisms, animal model studies suggested that maternal factors—including genetic variants like *PTEN* mutations or environmental exposures such as high-fat diet, or HFD might influence the bioavailability of aryl hydrocarbon receptor (AhR) ligands, for example, certain microbial metabolites derived from tryptophan metabolism through epigenetic reprogramming (Kirstein et al., 2021). This, in turn, could potentially impair fetal microglial functions, such as synaptic pruning. Preliminary research proposed the hypothesis that gut microbe-derived serotonin (5-HT) precursors might influence amygdala development via vagal nerve signaling pathways (Dinan and Cryan, 2017a; Spencer et al., 2024; Raskov et al., 2016). These cumulative findings underscore the important role of a “microbiota-immunity-neural” axis in neurodevelopment.

Animal models provided supportive evidence for exploring these potential mechanisms: the offsprings of GF dams exhibited deficits in axonal outgrowth, which could be ameliorated by butyrate supplementation in this model (Onyszkiewicz et al., 2019); in the maternal immune activation (MIA) model, blocking IL-17a signaling mitigated synaptic-associated gene expression abnormalities in offspring brains (Choi et al., 2016). However, these models and their derived conclusions face methodological limitations. Serum IL-8, a commonly studied systemic inflammation marker, showed weak correlations (R^2^ < 0.2) with local intestinal immune status, such as lamina propria lymphocyte composition (Bukys et al., 2024; Lammers-Lietz et al., 2022), challenging the direct extrapolation of peripheral markers to gut immunity. Furthermore, acute MIA models induced by high-dose LPS inadequately recapitulated the complexity of chronic, low-grade inflammation experienced during human pregnancy. Notably, in human studies, analyses primarily based on European populations may not capture potential heterogeneity in HLA (human leukocyte antigen)-microbiota interactions across different ethnic/racial groups (Van Dorp et al., 2014). Moreover, the influence of paternally derived epigenetic reprogramming on offspring immune system development remains insufficiently evidenced and is typically not systematically addressed in existing models (Soubry et al., 2014; Eggert et al., 2014). These methodological challenges complicate causal inference regarding specific metabolic pathways.

Explorations into gene-microbiota interactions are driving translational research on intervention strategies. Some studies suggested that combined assessment of maternal IL-10 levels and *Alcaligenaceae* abundance held potential for auxiliary ASD risk assessment models (Li et al., 2019), while maternal supplementation with AhR agonists such as indole-3-carbinol or specific probiotics aimed at promoting defined gut colonization has shown preliminary promise in early-phase clinical studies (Puccetti et al., 2022; Zhao et al., 2021). However, some bottlenecks must be overcome for effective personalized interventions: the development of higher spatiotemporal resolution immune cell profiling is needed to precisely distinguish functional IL-10 subtypes and their sites of action (Mangiola et al., 2024), and large-scale, multi-ethnic population cohorts are imperative to systematically validate the role of microbial metabolites within complex genetic contexts, such as HLA-restricted antigen presentation. Future intervention designs should rigorously incorporate multilayered interactions between individual genetic susceptibility backgrounds and environmental exposures to more effectively support fetal neuroimmune homeostasis (Dahoun et al., 2017; Suh et al., 2019).

### 3.4 Nutritional and metabolic factors

Epidemiological studies suggested a potential intergenerational link between maternal nutritional imbalance during pregnancy and altered offspring microbiota-gut-brain axis function. Maternal high-fat diet (MHFD) induces compositional shifts in gut microbiota, characterized by reduced abundance of Bacteroidetes phylum members associated with metabolic regulation, alongside over-representation of specific Clostridium species—*C. bolteae* and *C. histolyticum*—linked to metabolic dysfunction. This dysbiotic state correlates positively with increased risk of aASD in offspring. Diminished synthesis of SCFAs and concomitant increased LPS leakage potentially contribute, via induction of maternal-fetal interface inflammation, to the downregulation of key neurodevelopmental genes (e.g., BDNF, SHANK3). Evidence suggested this reduced expression might be attributed to epigenetic mechanisms (Esposito et al., 2023; Cermak et al., 2010). Notably, selective dietary preferences common in ASD children, such as low-fiber and high-sugar diets, may theoretically compound the effects of antenatal maternal dysbiosis, creating a cyclical interaction of metabolic and microbial imbalance that could exacerbate potential neurodevelopmental sequelae (Esposito et al., 2023; Cermak et al., 2010).

At the biological mechanism level, interactions between gut microbial metabolites and the maternal immune-fetal neural axis constitute a significant regulatory network. Butyrate deficiency, for instance, may impair blood–brain barrier integrity, indirectly activating microglia and disrupting synaptic pruning. MIA-induced inflammatory signals could inhibit differentiation of fetal dopaminergic neurons via Toll-like receptor 4 (TLR4)-dependent pathways (Islam et al., 2009). Concurrently, impairment of specific microbial functions, such as diminished folate biosynthesis capabilities of Bacteroides species and disruption of serotonin precursor metabolism primarily driven by Bifidobacterium (Engevik et al., 2021; Zheng et al., 2024), has been linked to impaired neocortical neuronal migration. Crucially, animal model studies indicated that the neurodevelopmental impact of dysbiosis was developmental-stage dependent: microbiota interventions administered prenatally, but not post-weaning, effectively reversed offspring autism-like behavioral phenotypes (Tartaglione et al., 2022), underscoring the importance of targeting critical developmental windows. However, mechanistic insights derived from models like germ-free mice warrant cautious extrapolation to the complex human physiological milieu.

Translational research indicated that antenatal supplementation with n-3 polyunsaturated fatty acids (PUFAs) or selected probiotics, in some studies, improved maternal microbiota diversity/composition alongside better offspring social behavior outcomes (Chen et al., 2019). Nevertheless, the significant methodological heterogeneity across existing clinical trials—concerning probiotic strains, intervention protocols, inclusion criteria, and outcome measures—substantially limits the generalizability of findings and their clinical translatability. For example, while germ-free murine models are vital for elucidating mechanisms such as microbiota-regulated synaptic protein expression, a critical limitation lies in their inability to fully recapitulate the multidimensional diet-microbiota-host genetics interactions inherent to humans. Furthermore, the absence of robust longitudinal data complicates efforts to disentangle the independent or synergistic effects of prenatal nutritional interventions from the influences of offspring postnatal microbial colonization dynamics. Future research necessitates integrating multi-omics approaches to precisely define key metabolic-epigenetic regulatory nodes. Utilizing more sophisticated animal models accommodating human microbiota, namely human microbiota-associated gnotobiotic models, is essential to overcome translational bottlenecks and move beyond broad-spectrum probiotics towards the development of strain-specific and function-targeted interventions.

## 4 Molecular mechanisms underlying the influence of maternal gut microbiota on offspring neurodevelopment

**Figure 2:**
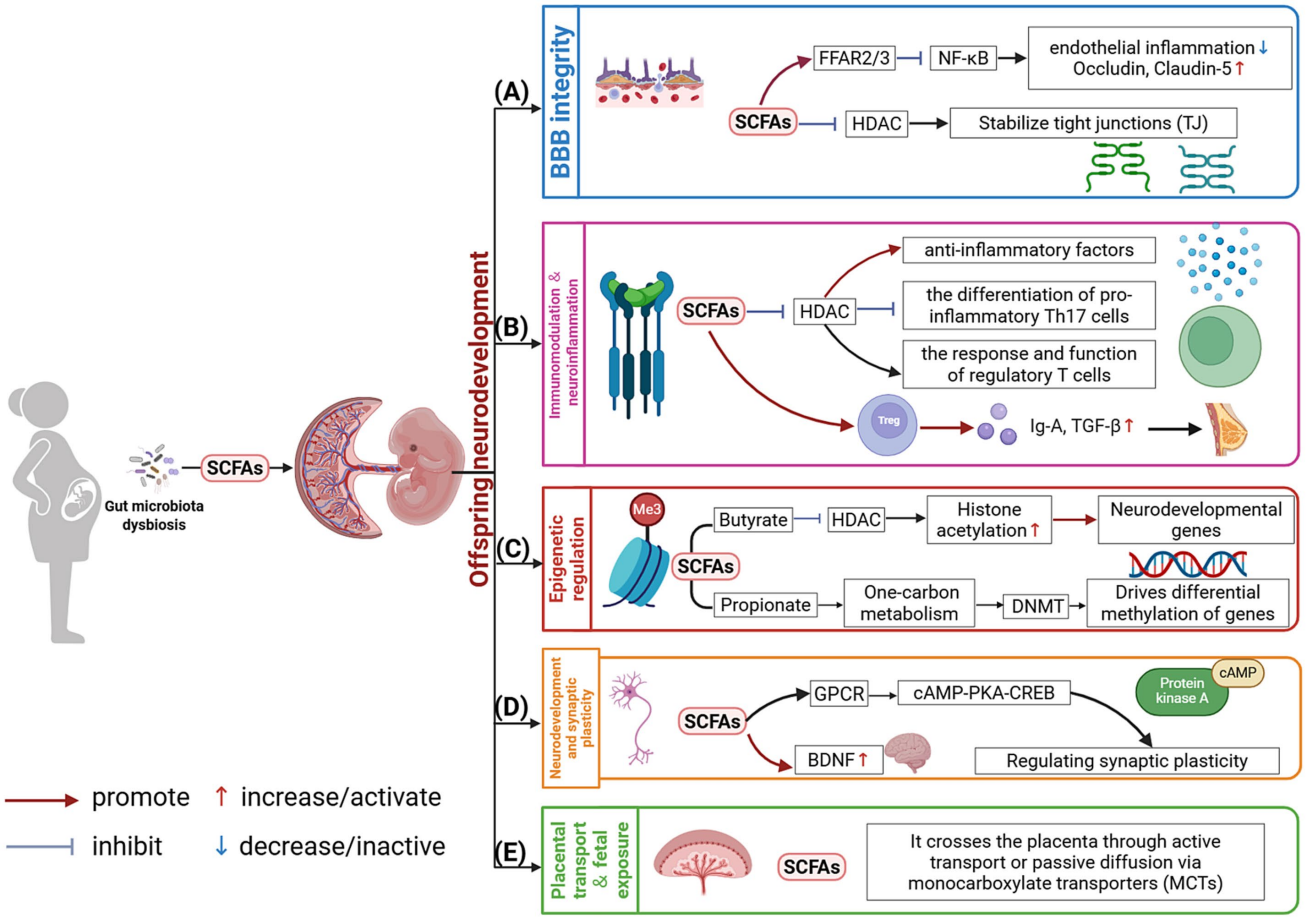
Multifaceted mechanisms of SCFAs in maternal gut microbiota-mediated programming of offspring neurodevelopment. Maternal gut microbiota-derived short-chain fatty acids (SCFAs) regulate offspring neurodevelopment through five core mechanisms: **(A)** Barrier enhancement: SCFAs (e.g., butyrate) strengthen blood–brain barrier (BBB) integrity by upregulating tight junction proteins (Claudin-5, Occludin) and inhibiting nuclear factor kappa-B (NF-κB) inflammatory signaling; **(B)** Immune modulation: They induce anti-inflammatory cytokines and regulatory T cell (Treg) differentiation, fostering a neuroprotective immune microenvironment; **(C)** Epigenetic regulation: As histone deacetylase inhibitors (HDACi) and one-carbon metabolic modulators, SCFAs control histone acetylation and DNA methylation of neurodevelopmental genes (e.g., brain-derived neurotrophic factor, BDNF); **(D)** Signaling activation: SCFAs trigger neuronal G protein-coupled receptor 41/43 (GPR41/43)-cyclic adenosine monophosphate (cAMP) and tropomyosin receptor kinase B (TrkB)-mechanistic target of rapamycin (mTOR) pathways, directing axonal outgrowth and synaptic pruning; **(E)** Placental transport: The illustration depicts transplacental SCFA transfer via specific monocarboxylate transporters (MCTs) from maternal circulation to the fetus. These interconnected mechanisms highlight the complexity of prenatal microbial programming (Created in BioRender; Xie, 2025, https://BioRender.com/2ksz0di).

To understand the roles of maternal gut microbiota in the offspring neurodevelopment, an abundance of researches has been performed and several molecular mechanisms have been elucidated (Figure 2).

### 4.1 Impairment of blood–brain barrier integrity as an underlying mechanism

Experimental models, particularly GF animal models, have demonstrated that the maternal gut microbiota fundamentally programs the development of the offspring’s BBB. Compared to offspring of normally raised dams, offspring exposed to a GF environment *in utero* exhibited significantly increased BBB permeability and a corresponding significant decrease of approximately 30–40% in the transcription of key tight junction (TJ) core proteins, such as occludin and claudin-5 (Gong et al., 2008). Studies found that gut microbiota status influenced BBB plasticity, as FMT into adult GF mice partially reversed BBB hyperpermeability, with leakage decreasing by approximately 25% (Gong et al., 2008). This microbial programming occurs, in part, via microbiota-host metabolic-immune crosstalk. Microbiota-derived SCFAs impacted downstream molecules of the NF-κB pathway by activating free fatty acid receptors (e.g., FFAR3), suppressing endothelial inflammation and potentially enhancing TJ stability. Conversely, pro-inflammatory microbial products, such as LPS, likely impaired TJ integrity via TLR4 signaling (Gong et al., 2008). The placenta acts as a critical barrier with selective transfer mechanisms. Direct evidence indicated that the human placenta efficiently transported SCFAs, such as acetate (Krajmalnik-Brown et al., 2015), while partially clearing larger molecules like LPS from the maternal circulation via CD14-mediated mechanisms (Faulk et al., 1987). This sculpts a relatively favorable intrauterine microenvironment for robust fetal BBB development.

Immune dysregulation and sustained inflammation driven by gut dysbiosis in the mother are key factors in disrupting BBB integrity. Animal models of maternal dysbiosis showed that dysbiosis potentiated T helper 17 (Th17) cell responses and differentiation, leading to significantly elevated circulating levels of pro-inflammatory cytokines. This subsequently induced phosphorylation and activation of signal transducer and activator of transcription 3 (STAT3), which suppresses expression of critical TJ proteins such as ZO-1and claudin-5 (Honda and Littman, 2012). Concurrent neutrophil activation enhanced matrix metalloproteinase-9 (MMP-9) activity and reactive oxygen species (ROS) production, with reported increases of around 70%, which contributed to vascular basement membrane degradation and endothelial injury (De Bondt et al., 2020). BBB dysfunction may link to neuropsychiatric disorders. While human studies reported inconsistent results, animal models supported the notion that BBB structural abnormalities could precipitate behavioral deficits. Specifically, developmental BBB defects caused by deficient Sonic hedgehog (Shh)/Wnt signaling pathway regulation in the endothelium induced ASD-like stereotyped behaviors in endothelial cell-specific Shh knockout models. This suggests a possible causal relationship between BBB structural abnormalities and neurobehavioral phenotypes, although its significance in humans requires further elucidation.

A multidimensional challenge for understanding microbiota-BBB interactions persists. First, SCFA effects are complex and concentration-dependent; for instance, propionate enhanced TJ protein expression at low concentrations via FFAR2 but may promote inflammation at high concentrations via FFAR3 (Han K. et al., 2024). Second, probiotic interventions often exhibit strain-specific effects, demanding systematic dissection of precise molecular networks. Clinical translation faces obstacles, including: significant species differences—despite high sequence homology for key TJ molecules like claudin-5—in regulatory networks and expression profiles; and limitations of *in vitro* BBB models whose transendothelial electrical resistance (TEER) values typically fall well below physiological levels (many achieve only ~50% or less). Furthermore, insufficient research addresses individual heterogeneity, such as sex differences—where BBB integrity decline in specific aging or disease models like Alzheimer’s models may occur earlier or faster in males compared to females—and critical window sensitivity, whereby the impact of perinatal interventions on neurodevelopmental risk often exceeds that of adult interventions. Future research necessitates integration of spatiotemporal dynamics and individualized variables, such as genetic background, sex and age, to build refined pathogenic mechanisms and interventional frameworks.

### 4.2 Neurodevelopmental regulation by microbial metabolites

The maternal gut microbiota can directly or indirectly regulate fetal brain development through the production of diverse metabolites (Tian et al., 2023). Core mechanisms primarily include three aspects: microbial synthesis or modulation of neurotransmitters and their precursors; mediation of epigenetic modifications; and regulation of maternal-fetal barrier function and its interactions (Brown et al., 2003; Bromer et al., 2013; Greene et al., 2019).

Taking SCFAs, as an example, these metabolites can cross the placenta via monocarboxylate transporters (MCTs) and enter the fetal circulation. Within the fetal brain, SCFAs activate G protein-coupled receptors (e.g., GPR41/43) on neurons, thereby modulating synaptic plasticity through the cAMP-PKA-CREB signaling pathway. Butyrate, as a HDAC inhibitor, upregulates the expression of key neurodevelopmental genes (e.g., *BDNF*) by elevating histone acetylation levels in their promoter regions. Animal model studies substantiated this: offspring of germ-free dams exhibited downregulated expression of axonogenesis-related genes (e.g., *Ntn1*, *Dcc*) and impaired function of BBB tight junction proteins (e.g., occludin). Exogenous SCFA supplementation partially rescued these phenotypes (Bromer et al., 2013; Greene et al., 2019), underscoring the potential significance of SCFAs in neurodevelopment.

Beyond SCFAs, other gut microbial metabolites participate in neurodevelopmental regulation. For instance, the tryptophan-derived metabolite indole-3-propionic acid (IPA) acted as a ligand for the AhR to modulate synaptic pruning by microglia. Certain microbiota-derived metabolites may also confer indirect neuroprotection by inhibiting *β*-amyloid aggregation (Dong and Perdew, 2020). The vertical transmission and functional impact of such metabolites depend on complex transport and signal integration mechanisms. While SCFAs can partially diffuse passively across the placenta (Ilyés et al., 2023), secondary bile acids regulated neuroinflammation by activating TGR5 receptors on placental and fetal tissues, synergizing with immune mediators at the maternal-placental interface to influence fetal neuroimmune homeostasis (Wang et al., 2011).

Epigenetically, butyrate-mediated histone acetylation alterations in the placenta promote neuronal differentiation in specific brain regions such as hippocampus (Jaju Bhattad et al., 2020; Levenson et al., 2004). Propionate may protect the developing BBB against oxidative stress by activating the Nrf2 antioxidant pathway (González-Bosch et al., 2021). Notably, rodent models indicated concentration-dependent effects of SCFAs on BBB permeability (Braniste et al., 2014). However, human analyses reveal that physiological SCFA concentrations in fetal brain tissue were significantly lower than exogenous doses required for interventions in animal models (Generoso et al., 2021). This pronounced species difference necessitates careful evaluation of metabolic kinetics in translational research.

Human cohort studies provide epidemiological support for associations between specific microbial metabolites and offspring neurodevelopmental outcomes (Moreau et al., 2019; Padilha et al., 2025). For example, prospective observational studies reported a positive correlation between maternal fecal butyrate levels during pregnancy and specific language scores in offspring (Hernández-Martínez et al., 2022; Barbian et al., 2022; Han W. et al., 2024); other studies describe abnormalities in levels of neuroactive metabolites and abundance of butyrate-producing bacteria in the gut microbiota of women at high risk of bearing children with ASD (Ahmed et al., 2022; Chen et al., 2020; Descamps et al., 2019). Nevertheless, establishing definitive causality remains challenging. Limitations arise from inherent constraints in metabolome-transcriptome correlation analyses, such as difficulties in distinguishing causal relationships from correlative changes or identifying dominant drivers (Sen et al., 2023; Paternain and Campion, 2013), as well as from confounding factors, and physiological or dosage differences between humans and animal models. Existing evidence also contains inconsistencies; for instance, the relationship between maternal plasma trimethylamine N-oxide (TMAO) levels and offspring ASD risk was debated across studies (Quan et al., 2020), reflecting the critical influence of confounders like ethnicity and diet on TMAO metabolic pathways and biological effects.

Future investigations necessitate integrative approaches to dissect causal mechanisms and guide interventions. Conditional gene knockout techniques in animal models, coupled with interventions during specific gestational time windows (Zhang et al., 2012; Nishimura et al., 2020), could precisely delineate the dynamic sensitivity of critical fetal brain developmental periods to distinct microbiota-derived metabolites (Faulk et al., 1987; Schirmbeck et al., 2022). Concurrently, quantifying the effects of sex dimorphism—such as the observed potential advantage in male fetal responsiveness to the microbial metabolite queuine (Queuine) (Faulk et al., 1987; Kokko, 2008)—on heterogeneity in neuroprotection or susceptibility mechanisms is required.

### 4.3 Dysregulated immune activation and neuroinflammation

Maternal gut probiotics such as *Lactobacillus* and *Bifidobacterium* may enhance gut barrier integrity and induce an anti-inflammatory milieu by activating pattern recognition receptors including TLRs in intestinal epithelial cells and through microbial metabolites such as SCFAs. SCFAs epigenetically modulate immune responses by inhibiting HDACs, thereby promoting anti-inflammatory cytokines and suppressing Th17 cell differentiation. Studies in germ-free mouse models indicated that supplementation with specific strains (e.g., *Lactobacillus reuteri*) elevated TGF-*β* and IgA levels in breast milk, subsequently inducing Treg differentiation in offspring (Alsharairi, 2023; Leser and Baker, 2024). However, this effect demonstrated strain-specific and population-dependent variations: some human studies report fluctuations in breast milk IgA levels, though strain-specific impacts require further clinical validation (Leser and Baker, 2024).

During MIA, hyperactivation of the Th17/IL-17A axis may increase placental permeability, facilitating fetal translocation of maternal proinflammatory cytokines such as IL-6. Current evidence suggested (*though not directly confirms*) that IL-6 suppressed fetal insulin-like growth factor 1 (IGF1) signaling via the placental JAK/STAT3 pathway while upregulating local proinflammatory proteins (Hsiao and Patterson, 2011). Animal experiments demonstrated that the blockade of placental IL-6 signaling mitigated MIA-associated offspring neurodevelopmental abnormalities; however, causal links between placental IL-6 and fetal brain pathology warrant further investigation (Wu et al., 2017). Clinical cohort studies revealed positive correlations between elevated maternal serum IL-6 and increased proinflammatory factors (e.g., IL-8, CXCL1) in umbilical cord blood, indicating a fetal proinflammatory microenvironment (Wu et al., 2017). Under extreme inflammation, such microenvironments may activate fetal microglia and increase neuronal injury risk (Yanowitz et al., 2002). Excess IL-6 may additionally inhibit brain-derived neurotrophic factor (BDNF) signaling, while IL-17A could disrupt post-translational modifications of neurodevelopmental proteins. Postmortem studies of ASD patients show elevated proinflammatory markers, microglial activation, and reduced neuronal density in the brain (Voineagu et al., 2011; Kim et al., 2017); however, causal attribution to prenatal immune exposure requires cautious interpretation.

Although specific probiotic formulations exhibit neuroprotective potential in animal models, translating these findings to humans shows substantial heterogeneity (Wang et al., 2016). For instance, *Lactobacillus reuteri* improves synaptic pruning defects in rodents but remains unconfirmed in human trials. *Bacteroides fragilis* ameliorated social behavior deficits only in subpopulations with elevated inflammatory markers, highlighting the importance of strain specificity and host immune context (Buffington et al., 2016; Hsiao et al., 2013). This translational gap likely arises from: (i) weak correlation between peripheral inflammatory markers and central nervous system pathology, and (ii) the complexities of host immunity represent a significant consideration; for instance, confounding factors involving a Th2 bias in type I diabetes pregnancies may contribute to the risk of ASD. Future research should integrate single-cell placental transcriptomics with functional cerebral organoids to dissect dynamic crosstalk between SCFAs and immune pathways, thereby defining spatiotemporally precise windows for probiotic interventions.

### 4.4 Epigenetic modifications and gene expression regulation

Maternal gut microbiota-derived metabolites have been demonstrated to possess the potential to modulate the activity of key host epigenetic enzymes. This modulation may consequently affect the spatiotemporal expression of genes critical for neurodevelopment. SCFAs are among the core effectors. In animal model studies, butyrate was shown to significantly inhibit the activity of histone deacetylases HDAC1, HDAC2, and HDAC3, leading to elevated acetylation levels at histone H3K9 and H4K16 residues in neuronal precursor cells (Korsten et al., 2023; Večeřa et al., 2018). This was concomitant with a marked increase in the transcriptional activity of the *Bdnf* gene (McClung and Nestler, 2008).

Conversely, propionate, a critical contributor to one-carbon metabolism, impacted DNA methylation by modulating methyl donor pools (Wu et al., 2023). Mechanistic studies suggested that it may enhance the activity of DNA methyltransferases (DNMTs), thereby driving differential methylation patterns of the imprinted gene *Igf2* in placental and fetal brain tissue (Wang et al., 2013; Steegers-Theunissen et al., 2009), which probably disrupted the proliferation/differentiation balance in neural precursor cells (Latchney et al., 2011).

Other metabolites, including specific vitamins such as folic acid, vitamin B12, as well as bile acids, may interact synergistically or antagonistically with SCFA pathways. For instance, folic acid, synthesized by microbiota including specific *Bifidobacterium* strains, served as an essential cofactor in the DNA methylation cycle (Pompei et al., 2007). It helped maintain a hypomethylation state at promoters of genes vital for neural tube development such as *Pax3* (Song et al., 2015). Observational evidence consistently indicated that low maternal folate levels during pregnancy significantly increased the risk of neural tube defects in offspring (pooled OR = 2.4, 95% CI: 1.6–3.7).

In contrast, the secondary bile acid deoxycholic acid (DCA) was observed in some experimental models to activate the FXR-nuclear receptor-mediated HDAC3 pathway, significantly suppressing *Shh* gene expression in the cerebellum (Struhl, 1998; Chiang, 2013). This effect may involve decreased histone acetylation at its promoter region, potentially impacting nervous system development. It is noted that the folic acid synthesis capability is strain-specific among *Bifidobacteria* (Sugahara et al., 2015).

Crucial neurodevelopmental genes such as *BDNF* and *MECP2* are subject to highly dynamic and complex epigenetic regulation (Allison et al., 2021). Maternal microbial metabolites bidirectionally modulate these genes by altering DNA methylation and histone modification states. For example, maternal gut microbiota dysbiosis induced by a high-fat diet correlated significantly with hypermethylation at the CpG dinucleotide within exon IV of the *Bdnf* gene in the offspring hippocampus in mouse models (Kimura et al., 2020). Conversely, butyrate supplementation restored phosphorylation levels of Methyl-CpG-binding protein 2 (MeCP2) in specific animal models, potentially alleviating its repression of *Bdnf* transcription. The functional consequences of DNA methylation are highly context-dependent: MeCP2 binding to the methylated CpG island at the *Dlx5* gene locus, involved in GABAergic neuronal differentiation, resulted in repression (Chen et al., 2003). Maternal probiotic intervention reduced methylation levels at specific *Dlx5* CpG sites in mouse models, concomitant with mitigated impairment of GABAergic neuronal differentiation (Liao et al., 2023). Single-cell epigenomic analyses have further indicated that aberrant methylation at non-CpG sites, such as CHH trinucleotides, may be associated with alterations in chromatin spatial conformation—a phenomenon observed in approximately 30% of ASD animal models—and could independently influence synaptic pruning. Nevertheless, the precise regulatory mechanisms remain unvalidated.

Current research underscores sophisticated interactions between specific strain-derived metabolites and the host epigenetic system. Translating these findings presents substantial challenges. Clinical cohort analyses report that hypermethylation of the *OXTR* (Oxytocin Receptor) gene in peripheral blood of ASD children inversely correlated with maternal *Prevotella* abundance (Andari et al., 2020). This remains an observational association. Reference suggests the probiotic strain *Lactobacillus reuteri* may induce hypomethylation at the *Foxp3* locus, a key transcription factor for regulatory T cells, potentially via secretion of bioactive folate forms like 5-methyltetrahydrofolate, thereby mitigating neuroinflammation risk.

However, significant limitations persist: foundational studies often rely on broad-spectrum antibiotic-induced microbiota depletion models or direct metabolite injection/gavage, poorly replicating the spatiotemporal heterogeneity and metabolic dynamics present within a natural gut microbiota network (Gheorghe et al., 2021; Mao et al., 2022). Furthermore, observed associations between specific histone modifications and metabolite abundances in human populations lack established causality. Future research must integrate data on placental and BBB transport dynamics of microbial metabolites with high-resolution single-cell epigenomics to systematically delineate strain-host interactions within specific tissues and developmental timepoints, overcoming mechanistic fragmentation and advancing microbiota-based epigenetic intervention strategies (Duck and Connor, 2016).

### 4.5 Neuronal connectivity and signaling pathway interference

Maternal gut microbiota could potentially regulate fetal neural circuit development—including neurite formation and refinement—through microbiota-derived metabolites acting on signaling pathways. Current evidence indicated limited placental penetration capacity of specific microbial metabolites (Kolahi et al., 2018). These compounds may modulate critical neuronal signaling pathways such as AKT/mTOR and Wnt/*β*-catenin in the developing fetal central nervous system (Li et al., 2023).

In GF mouse models, researchers observed impaired axonal development—manifested as reduced axonal length—compared to conventionally colonized controls. This impairment correlated with dysregulated expression of axon guidance molecules. Exogenous supplementation with microbiota metabolites (e.g., IPA or 4-ethylphenylsulfate [4-EPS]) was found to improve axonal branching complexity under specific conditions (MacKay et al., 2024). This effect may partially involve β-catenin stability regulation or nuclear translocation.

Furthermore, bifidobacterial colonization in murine models downregulated genes associated with excessive synaptogenesis (Wang et al., 2014). This colonization concomitantly alleviated abnormal hippocampal synapse density elevation observed in GF models. These findings suggest microbiota metabolites may regulate synaptic developmental processes and influence microglia-mediated synaptic pruning, though their effects demonstrate bidirectional modulation.

Maternal dysbiosis during gestation may disrupt key neurodevelopmental signaling pathways, impairing normal neural circuit establishment. Evidence suggested microbiota imbalances affect Wnt signaling activity. In specific models, altered expression of cell cycle-related genes was observed in the fetal prefrontal cortex, potentially disrupting neurodevelopmental trajectories (Li et al., 2023). Interventions with particular bifidobacterial strains were reported to restore Wnt/*β*-catenin pathway equilibrium in corresponding models.

The BDNF-tropomyosin receptor kinase B (TrkB) pathway also exhibited microbiota connections (Sudo et al., 2004). Maternal probiotic administration elevated BDNF expression in offspring hippocampi across multiple studies. This upregulation was postulated to enhance dendritic spine maturation via downstream signaling activation, coinciding with behavioral improvements such as reduced social avoidance in male offspring. Conversely, GF models demonstrated diminished cortical BDNF/mTOR axis activity, accompanied by synaptic transmission deficits (e.g., reduced synaptic vesicle release frequency). These anomalies align with inhibitory synaptic defects [e.g., decreased miniature inhibitory postsynaptic current (mIPSC) amplitude] in neurodevelopmental disorder models.

However, several key challenges persist in understanding the maternal microbiota-fetal neurodevelopment axis. The regulatory processes exhibit significant spatiotemporal heterogeneity, and interspecies physiological differences—placental barrier permeability and metabolic capacity—hinder clinical translation. Notably, reported discrepancies in indole metabolite processing between humans and rodents require rigorous validation. Future studies should integrate multi-omics data, including metagenomics, metabolomics, epigenomics, and employ high spatiotemporal-resolution techniques, for instance, single-cell multi-omics coupled with live imaging, to dissect key pathways like the vagus-immune-metabolic axis.

## 5 Role of probiotics in preventing offspring autism

### 5.1 Mechanistic links

Emerging evidence indicates characteristic gut microbial dysbiosis in children with ASD, typically manifesting as reduced microbial diversity (Kang et al., 2013). While multiple studies report decreased Prevotella abundance and enriched Clostridium populations, a 2024 conference abstract documented contrasting findings (Prevotella and Clostridium enrichment), suggesting microbiome profiles may be influenced by geographical variations, cohort heterogeneity, or methodological factors (Debelius et al., 2016; Chu et al., 2024; Sun et al., 2020). The generalizability of these signatures requires large-scale validation.

Animal models provide critical evidence for the gut-microbiota-brain axis: Fecal microbiota transplantation from ASD donors to germ-free mice induces social deficits in recipients, with the microbial metabolite p-cresol identified as a key mediator of such behavioral abnormalities (Bermudez-Martin et al., 2021). Mechanisms linking dysbiosis to neurodevelopmental disruption involve multi-pathway crosstalk. Immunologically, maternal dysbiosis promotes Th17 cell differentiation and amplifies IL-17a production; this cytokine—transferred placentally or locally produced—directly impairs fetal cortical neuron migration (Choi et al., 2016). Metabolically, SCFA deficiency inhibits hippocampal synaptic plasticity via suppressed HDAC activity, whereas reduced levels of TMAO, a positive neurodevelopmental regulator, correlate with developmental impairments (Tang and Li, 2021; Li et al., 2018). Epigenetically, maternal high-fat diets alter microbiota-derived metabolites, modulating DNA methylation patterns in the placenta and fetal brain, thereby regulating expression of neurodevelopmental genes like BDNF (Martinowich et al., 2003; D’Aquila et al., 2020; Keleher et al., 2018).

Maternal gut microbiota may influence offspring neurodevelopment through vertical transmission; although elevated Alcaligenaceae abundance occurs in ASD children, direct evidence establishing causal links between maternal transmission of this taxon and ASD risk is lacking, and the mechanistic role of maternal-specific microbial shifts requires further validation (Cook and Prinz, 2022). Hypotheses regarding conserved “core microbiota signatures” across populations face limitations, as stability observed in plant-microbe systems cannot be directly extrapolated to human ASD studies; human gut microbiota heterogeneity is dynamically shaped by diet, antibiotic exposure, and socioeconomic status, challenging the universality of putative core microbial features (West et al., 2022).

Maternal intervention studies offer critical insights: In MIA models, probiotic treatment (*Bacteroides fragilis*) ameliorates offspring neurobehavioral deficits, potentially via central GABA receptor modulation or SCFA metabolic pathway restoration (Hsiao et al., 2013; Sharon et al., 2019). Current evidence supports two non-mutually exclusive hypotheses, yet their robustness warrants cautious interpretation (Vuong and Hsiao, 2017; Cryan et al., 2019). The Vertical Transmission Hypothesis posits microbial inheritance, yet evidence remains limited in complex human microbiomes; microbes and metabolites may indirectly affect neurodevelopment via birth mode or breastfeeding, but causal chains linking specific taxa transmission to ASD pathogenesis have not been established (Ferretti et al., 2018; Aatsinki et al., 2019; Sordillo et al., 2019). The Critical Window Intervention Hypothesis suggests perinatal probiotic supplementation exerts neuroprotective effects in animals, and observational studies suggest potential neurodevelopmental benefits; nonetheless, human translation faces challenges, as animal models incompletely recapitulate gene–environment interactions, and human studies are confounded by unmeasured variables (Buffington et al., 2016; Slykerman et al., 2017; Dinan and Cryan, 2017b).

Future research should integrate large prospective cohorts with multi-omics approaches and develop humanized animal models or conditional knockout systems to address: (1) causality and molecular mechanisms of maternal-offspring microbial transmission; (2) reliability of ASD-associated “core microbiota signatures” across diverse populations; and (3) efficacy and safety of microbiota-targeted interventions in human clinical translation.

### 5.2 Clinical efficacy of prenatal probiotic intervention

#### 5.2.1 Current evidence gap

Robust evidence from large-scale, high-quality RCTs or prospective cohort studies is currently lacking to support the universal efficacy of prenatal probiotic supplementation in reducing ASD risk in offspring.

#### 5.2.2 Animal model findings

Rodent studies have indicated that specific probiotic strains, such as select Lactobacillus spp., may improve offspring neurobehavioral phenotypes, including reduced stereotyped behaviors and enhanced social interaction, while also decreasing neuroinflammatory markers; such effects are hypothesized to occur via modulation of the gut-brain axis, although the clinical translatability of these findings requires cautious interpretation.

#### 5.2.3 Limitations in human studies

Direct investigation of prenatal probiotics for preventing offspring ASD is scarce, with existing studies typically limited by small sample sizes; most human data focus on probiotic administration to children with established ASD diagnoses, and results from such interventions show mixed efficacy and frequently lack statistical significance, indicating that further investigation is warranted (Zyoud et al., 2023). Current clinical research on autism spectrum disorder (ASD) primarily focuses on interventions targeting the gut microbiota, including probiotics, prebiotics, synbiotics, fecal microbiota transplantation (FMT), microbiota transfer therapy, dietary interventions, as well as antibiotic and antifungal therapies. Although many studies report symptomatic improvements following these interventions, their conclusions remain limited due to challenges in establishing causality, generally small cohort sizes, and a limited number of available studies. Thus, further large-scale and rigorously designed studies are warranted to validate the clinical efficacy and mechanisms of these interventions (Liu, 2022; Buffington et al., 2016; Burket et al., 2013; Callery and Geelhaar, 1985; Caporaso et al., 2010).

#### 5.2.4 Indirect effects and heterogeneity

Indirect evidence suggests that prenatal probiotics may influence neurodevelopmental trajectories by mitigating maternal complications such as gestational diabetes mellitus (GDM) (Appleton, 2018; Barthow et al., 2016; Kijmanawat et al., 2019). Nevertheless, a definitive causal pathway linking GDM mitigation via probiotics to ASD risk reduction remains unestablished (Berding and Donovan, 2016; Cryan et al., 2019). Major sources of inconsistency include probiotic strain selection, dose variability, and timing and duration of administration (Kristensen et al., 2016; McFarland et al., 2018). Given the high genetic heterogeneity and multifactorial etiology of ASD, probiotics may offer benefits only in specific high-risk subpopulations, particularly individuals with baseline gut dysbiosis or carriers of neurodevelopmental disorder-associated genetic variants (Ronald and Hoekstra, 2011; Kang et al., 2019; Sharon et al., 2019).

#### 5.2.5 Proposed mechanisms

Two mechanistic hypotheses potentially explain the neuroprotective effects. First, the Gut-Brain Axis Modulation hypothesis posits that maternal gut dysbiosis linked to ASD disrupts fetal neurodevelopment via altered production of microbial metabolites, modified synthesis of neuroactive compounds, and induction of systemic low-grade inflammation (Hsiao et al., 2012; Stilling et al., 2016; O’Mahony et al., 2015). Probiotics might remodel the maternal gut microbiota, thereby potentially influencing fetal gut-immune-neural circuit development (El Aidy et al., 2012; Vuong et al., 2020). Animal models further suggest that probiotics exert strain-specific effects on HPA axis regulation and maternal metabolic homeostasis, potentially mitigating adverse impacts on hippocampal neurogenesis (Sgritta et al., 2019; Möhle et al., 2016). Second, the Vertical Transmission Hypothesis proposes that maternal microbes and/or their metabolites program fetal epigenetic landscapes, subsequently shaping immune maturation and neural plasticity (Gomez de Agüero et al., 2016; Thion et al., 2018b). It is important to note that these mechanisms derive predominantly from preclinical models; rigorous experimental validation in humans remains essential (Needham et al., 2021).

#### 5.2.6 Future research imperatives

Standardized study designs should incorporate uniform core parameters, including clinically validated probiotic formulations, defined dosages, standardized intervention windows, and consistent neurodevelopmental endpoints, while clinical trials should prioritize high-risk subgroups, such as carriers of neurodevelopmental copy number variants (CNVs) mutations or individuals with severe baseline dysbiosis, coupled with longitudinal cohorts for long-term offspring follow-up (Reid, 2016; McFarland et al., 2018; Zwaigenbaum et al., 2015; Vuong and Hsiao, 2017). Further, mechanistic studies should leverage advanced models, such as blood–brain barrier organoids and fetal brain organoid cocultures, to probe the direct effects of probiotic metabolites on blood–brain barrier function and synaptogenesis (Gabriel et al., 2020; Qian et al., 2019). Metagenomic-metabolomic correlative analyses may identify key maternal-fetal microbial and metabolite transfer hubs (Walker et al., 2017; Younge et al., 2019). Finally, integrating these mechanistic insights with real-world evidence will ultimately define clinical applicability, target populations, and risk–benefit ratios for ASD precision prevention (Geschwind and State, 2015; Kim et al., 2018; Courchesne et al., 2020).

## 6 Future perspectives

### 6.1 Systematic resolution of spatiotemporal specificity in gut microbiota-mediated neural development

Although current studies reveal associations between gut microbial metabolites and fetal neuronal axonogenesis, their precise impact on ASD-related neural circuit development remains unclear (Needham et al., 2021). This gap pertains particularly to dose–response dynamics and temporal mechanisms during critical gestational windows (Dunlop et al., 2015). Existing models exhibit critical limitations: broad-spectrum antibiotic perturbation fails to recapitulate complex host-microbiota interactions in human pregnancy, while cross-sectional studies cannot establish causality (Dunlop et al., 2015). Addressing this will require spatiotemporally precise intervention and monitoring tools. We will integrate CRISPR-associated protein 9 (CRISPR-Cas9)-mediated targeted editing of gut commensals with *in vivo* two-photon microscopy to establish gestational-stage-specific models (Dunlop et al., 2015). Such models will enable real-time tracking of how concentration gradients of specific microbial metabolites or precursors regulate axon guidance signaling pathways in vivo (Thion et al., 2018a). Implementing this strategy will necessitate: (i) applying advanced spatial metabolomics coupled with high-resolution mass spectrometry to enhance tissue-level metabolite localization; and (ii) performing three-dimensional imaging of intestinal crypt architectures to resolve spatial distributions of commensal microbes and metabolites within intestinal niches at submillimeter resolution. This integrated platform will resolve whether key developmental processes—such as embryonic neural crest cell migration—depend on spatially patterned microbial metabolic activities.

### 6.2 Integrated multi-omics approaches confront dual challenges of data heterogeneity and causal complexity

Research on microbiome-host interactions faces dual challenges: (i) Highly nonlinear relationships between high-dimensional microbial features and host physiological pathways impede critical regulatory insights from conventional linear models (Lloyd-Price et al., 2019); (ii) The limited resolution of 16S rRNA gene sequencing frequently fails to distinguish functionally active strains, potentially yielding spurious associations (Jovel et al., 2016). Current understanding of Bifidobacterium-mediated serotonin biosynthesis regulation, for instance, remains correlative, lacking mechanistic validation (Yano et al., 2015). To overcome these limitations, two innovative strategies have been proposed: constructing heterogeneous biological networks integrating microbe-metabolite-host gene nodes, within which graph neural networks (GNNs) with attention mechanisms could quantify regulatory weights and directionality for keystone species acting on host pathways within complex ecologies (Faust et al., 2012); and combining variational autoencoders (VAEs) with microbial ecological constraints to infer biologically plausible absolute abundances from relative abundance data, thereby mitigating compositional bias effects (Faust et al., 2012). For robust causal inference, frameworks beyond univariate Mendelian randomization (MR) are essential (Bowden et al., 2017). Future studies should implement structural equation modeling (SEM) incorporating ecological network dynamics. Such approaches could quantitatively validate observed microbiome-host relationships—as exemplified by Butyricicoccus members, which have experimentally demonstrated HDAC inhibition activity affecting host pathways like GABAergic neuron differentiation.

### 6.3 Intergenerational mechanistic validation requires humanized research paradigms

Human cohort studies face ethical constraints in controlling maternal diet-environment toxin interactions (Hanrahan et al., 1992). To address this and the evolutionary limitations of rodent models for gut-brain axis translation—alongside the absence of systematic immune-microbiota simulation in current human brain organoids—innovative solutions target two domains: confounder control and cross-platform integration (Quadrato et al., 2017). For confounder control, researchers should prioritize prospective cohort designs or standardize interventions, applying high-dimensional multivariate models to correct biases (Magnus et al., 2016). Cross-platform integration requires: (i) engineering 3D-bioprinted microfluidic systems that incorporate placental barrier organoids/chips within BSL-2 containment integrated with humanized living bacterial biofilms (Blundell et al., 2018); and (ii) establishing computational-experimental feedback loops via exposome models derived from mother-infant multi-omics data—including metagenomic, metabolomic, and epigenomic profiles—to predict targets, with subsequent ex vivo validation (Wild, 2012). Locally validated targets warrant development of gut-restricted delivery systems using pH-responsive chitosan coatings for oral gavage with site-specific release. Current achievable goals must focus on localized intestinal interventions—excluding systemic OMV delivery or maternal metabolic gradient simulation. This integrated strategy spanning bioinformatic prediction, multi-dimensional organoids, and engineered delivery provides a systematic framework to resolve prenatal microbiota-host networks underlying ASD risk (Hsiao et al., 2013).

## 7 Summary

Maternal gut dysbiosis is recognized as a significant non-independent environmental factor contributing to the risk of offspring ASD. Current understanding of its pathological mechanisms, primarily derived from preclinical studies, supports a multi-pathway synergistic model: Strong preclinical evidence indicates that dysbiosis triggers aberrant maternal immune activation, disrupts homeostasis of key metabolites, and potentially induces epigenetic reprogramming.

Collectively, these changes impair critical fetal neurodevelopmental processes in animal models, encompassing synaptogenesis, synaptic pruning, and the establishment and functional maturation of the BBB.

However, translating probiotic interventions into effective ASD prevention or treatment presents substantial challenges. Key scientific bottlenecks include heterogeneity in intervention efficacy due to strain-specific effects of probiotics and complexity in host-microbiome interactions; unresolved questions regarding maternal gut microbiota-derived metabolites in humans—specifically, their efficient placental transfer, post-transfer metabolic transformations, effective target-site concentrations, and precise mechanisms of action on fetal neurodevelopment; and limitations in existing human evidence supporting the pathogenic chain, which relies heavily on observational studies frequently confounded by covariates such as maternal diet, antibiotic usage, and environmental exposures, thereby compromising causal inference.

Consequently, it must be unequivocally emphasized that conclusive causal evidence linking maternal dysbiosis to altered fetal neurodevelopment and subsequent ASD pathogenesis in humans remains insufficient, and probiotic efficacy for ASD prevention or treatment lacks consistent validation through adequately powered and rigorously designed human RCTs.

Probiotics and fecal microbiota transplantation (FMT) show potential in modulating gut microbiota and alleviating symptoms of autism spectrum disorder (ASD). However, due to limited evidence quality, lack of standardization, and potential risks, they remain experimental therapies at present. Future large-scale randomized controlled trials (RCTs) are warranted to elucidate their efficacy mechanisms, establish individualized protocols, and rigorously monitor long-term safety.

To overcome current limitations and shift from associative findings to actionable target identification, future research must elucidate the spatiotemporal dynamics of dysbiosis-induced neurodevelopmental perturbations during critical gestational windows; integrate multi-omics data (e.g., metagenomics, metabolomics, and epigenomics) and employ advanced computational approaches to reconstruct and decode the “microbiota-metabolite-host gene/pathway” interaction network, enabling precise identification of core drivers and causal pathways; develop human-relevant gut-brain axis organoid models that better recapitulate human physiology; and design rigorous clinical trials to systematically validate mechanistic hypotheses and intervention strategies.
